# Novel acridone-modified MCM-41 type silica: Synthesis, characterization and fluorescence tuning

**DOI:** 10.3762/bjnano.2.33

**Published:** 2011-06-09

**Authors:** Maximilian Hemgesberg, Gunder Dörr, Yvonne Schmitt, Andreas Seifert, Zhou Zhou, Robin Klupp Taylor, Sarah Bay, Stefan Ernst, Markus Gerhards, Thomas J J Müller, Werner R Thiel

**Affiliations:** 1 TU Kaiserslautern, Fachbereich Chemie, Erwin-Schrödinger-Straße 52–54, D-67653 Kaiserslautern, Germany; 2 TU Chemnitz, Institut für Chemie, Straße der Nationen 62, D-09111 Chemnitz, Germany; 3 Friedrich-Alexander-Universität Erlangen-Nürnberg, Erlangen Catalysis Resource Center (ECRC), Egerlandstraße 3, D-91058 Erlangern, Germany; 4 Friedrich-Alexander-Universität Erlangen-Nürnberg, Institut für Partikeltechnologie, Cauerstraße 4, D-91058 Erlangern, Germany; 5 Heinrich-Heine-Universität Düsseldorf, Institut für Organische Chemie und Makromolekulare Chemie, Universitätsstraße 1, D-40225 Düsseldorf, Germany

**Keywords:** acridone, co-condensation, fluorescence, scandium, MCM-41

## Abstract

A Mobil Composition of Matter (MCM)-41 type mesoporous silica material containing *N*-propylacridone groups has been successfully prepared by co-condensation of an appropriate organic precursor with tetraethyl orthosilicate (TEOS) under alkaline sol–gel conditions. The resulting material was fully characterized by means of X-ray diffraction (XRD), N_2_-adsorption–desorption, transmission electron microscopy (TEM), IR and UV–vis spectroscopy, as well as ^29^Si and ^13^C CP-MAS NMR techniques. The material features a high inner surface area and a highly ordered two-dimensional hexagonal pore structure. The fluorescence properties of the organic chromophore can be tuned via complexation of its carbonyl group with scandium triflate, which makes the material a good candidate for solid state sensors and optics. The successful synthesis of highly ordered MCM materials through co-condensation was found to be dependent on the chemical interaction of the different precursors.

## Introduction

Mesoporous silicates are widely used for a variety of applications such as gas storage and heterogeneous catalysis, e.g., the synthesis of ε-caprolactam [1], or the decomposition of nitrous oxides [2]. MCM-41, MCM-48 and other silica materials can normally be functionalized either by in situ post-sol–gel modification or by direct co-condensation of different types of organic precursors [3]. The latter method often leads to a more homogeneous distribution of the desired functionalization within the material. It also provides the possibility to characterize application-tailored sol–gel precursors prior to implementing them into the solid, thus increasing the depth of information compared to the data solely drawn from solid state measurements.

Focusing on the synthesis of novel inorganic–organic hybrid materials, we also investigated new ways to produce trialkoxysilanes bearing polycyclic aromatic compounds as terminal groups, Ar–(CH_2_)*_n_*–Si(OR)_3_ (*n* = 3, R = Me, Et), which may lead to interesting optical or electronic properties [4–6]. Acridone, being a well-known fluorophore used, e.g., for chemosensors [7], has previously been reported to be suitable for the p*K*_a_ determination of lanthanide salts in aqueous solution, the fluorescence undergoing a bathochromic shift directly correlated to the acidity of the cation bound to its carbonyl group [8]. We therefore sought to create a micro- or mesoporous material containing covalently bound acridone units that would serve the same purpose, by choosing the amino function of acridone as the functional group to be modified. Trialkoxysilanes with a variety of functional groups have already been prepared by *N*-alkylation of amines using 3-iodopropyltrimethoxysilane (IPTMS) [9] or 3-bromopropyltrimethoxysilane (BPTMS) [10], by sulfamidation [11], by imide [12] or via imine forming reactions [13] using 3-aminopropyltriethoxysilane (APTES). In 2009, an *N*-alkylated acridone derivative bearing a (tri-isopropyloxysilyl)propyl group and its application as an anion-selective fluorescent probe were reported by Lin and Chen [14]. However, to the best of our knowledge, up to now, no MCM-like material featuring covalently bound acridone units has been described.

## Results and Discussion

**Scheme 1:**
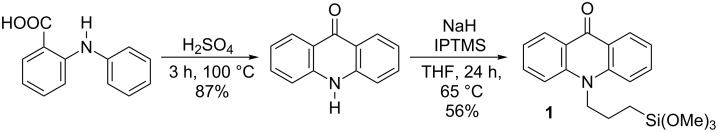
Synthesis of the sol–gel precursor **1**.

Compound **1** was prepared in a two-step sequence (Scheme 1): Following a published procedure, acridone was obtained from commercially available *N*-phenylanthranilic acid by an acid catalyzed ring closure reaction [15]. To attach the silyl functionalized linker, acridone was deprotonated by NaH and the resulting anion reacted with 3-iodopropyltrimethoxysilane (IPTMS).

Co-condensation of TEOS with organosilanes in the presence of an appropriate structure-directing template allows the direct and homogeneous incorporation of organic functionalities into a mesoporous material. Applying this method, we were recently able to introduce up to 30 wt % of redox-active phenothiazines into mesostructured silicas [4–6]. The resulting materials showed a continuous decrease of ordering with increasing bulk of the organic groups, although all exhibited very high specific surface areas. As the formation of micelles will strongly depend on the nature of the organic molecule and its concentration, it was decided to keep the amount of precursor **1** at 10 mol % with respect to TEOS in order to be sure that a highly ordered material would be obtained. The MCM-41 analogue was prepared by a modified synthesis previously reported by Pang et al. [15], in which aqueous ethylamine is used in order to adjust smoothly the pH-value of the solution. Given the fact that polycyclic compounds are rather bulky organic moieties which might behave differently when undergoing a sol–gel transformation, a slightly larger molecule (stearyltrimethylammonium bromide, C_18_TAB) was chosen as the templating agent.

However, obtaining materials with high surface areas has previously been shown to be also dependent on the boiling point of the sol–gel solution. Thus, the addition of larger amounts of volatile organic solvents such as THF to the aqueous phase often prevents the formation of highly ordered mesoporous structures [16]. On the other hand, the π-stacking of large aromatics generally causes them to be less readily dissolved and requires larger amounts of solvents, which are mostly immiscible with water. Therefore, pure TEOS was tested as a mediating agent for introduction into the sol–gel process. It turned out to be beneficial for the co-condensation process that precursor **1** could be mixed with TEOS rather easily, although it did not prove possible to obtain a homogeneous solution. Despite the fact that it was rather difficult to handle by syringe, a mixture of TEOS and **1** could be converted to the corresponding mesostructured silica **MCM-ACR**.

In contrast to **1**, a similar compound prepared from pyrenesulfonyl chloride and APTES via sulfamidation immediately yielded a flocculent precipitate when brought in contact with TEOS. This observation might be explained by the combination of the pyrene moieties, forming strong π-bonding interactions, and the highly polar hydrogen bonding sulfonamide, causing the compound to become inhomogeneous in TEOS. As expected, the material obtained from the pyrene precursor showed a drastically decreased inner surface area of only 322 m^2^·g^−1^ and no ordered material structure at all.

The CHNS analysis of **MCM-ACR** clearly indicates a slightly increased ratio between the dye and silica (1:7.5) compared to the initial ratio of the synthesis (1:9), probably resulting from different rates of hydrolysis of TEOS and the RSi(OMe)_3_ groups. Converting the materials’ molecular composition to the amount of *N*-propylacridone moieties per gram, we obtained a dye loading of approximately 240 mg·g^−1^.

**Figure 1:**
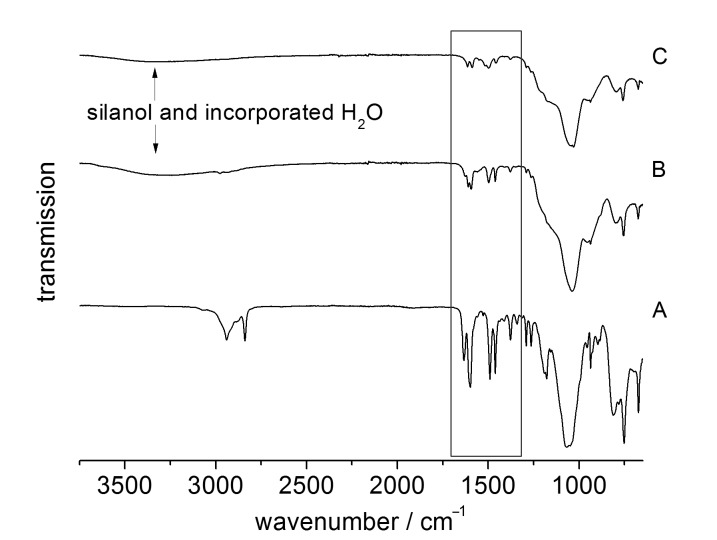
Infrared spectra of compound **1** (A), **MCM-ACR** (B) and **MCM-ACR** + Sc(OTf)_3_ (C). The box marks the section given in Figure 2 (resolution ±2 cm^−1^).

From the infrared spectra of **1** and both the unmodified as well as the modified **MCM-ACR**, the aromatic and aliphatic C–H vibrational bands around 3000 cm^−1^ showed reduced intensities in the solid and were mostly covered by the weak, yet broad, absorption bands of the silanol groups and bands from water trapped in the silica framework (Figure 1).

**Figure 2:**
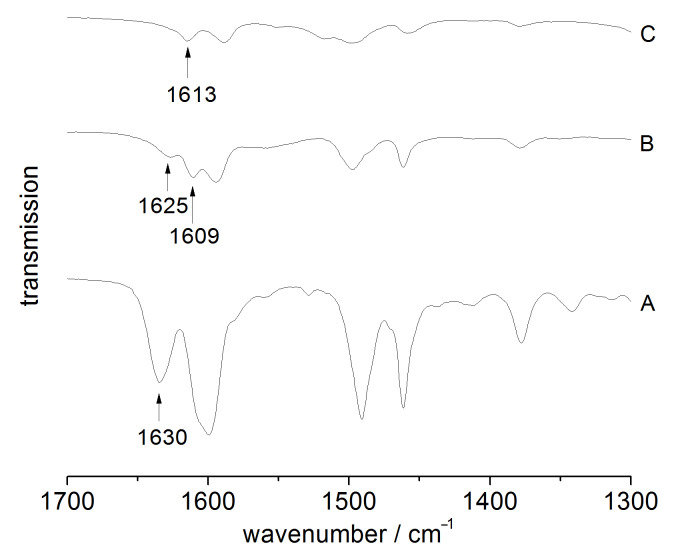
C=O vibrational band section of the infrared spectra of compound **1** (A), **MCM-ACR** (B) and **MCM-ACR** + Sc(OTf)_3_ (C).

The spectra were also found to be in accordance with the interpretation of the infrared spectrum of free acridone reported earlier by Berezin et al. [17]. By comparing the region between 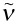 = 1700 cm^−1^ and 1300 cm^−1^, complex formation with the scandium(III) cation is clearly observed. The infrared absorption of the C=O vibrational band (precursor **1**: 1630 cm^−1^, *N*-methylacridone: 1630 cm^−1^ [18]) shifts slightly to lower wavenumbers (Figure 2) after the immobilization, and also splits into two resonances (**MCM-ACR**: 1625 cm^−1^, 1609 cm^−1^) indicating an interaction of the carbonyl group with Lewis or Brønsted acidic or with hydrogen-bond-donating surface sites, which has previously been described for fluorescent probes such as Michler’s ketone [19]. This again changes after the reaction with Sc(OTf)_3_: A single C=O absorption emerges at 1613 cm^−1^. The redshift of the absorption frequency is conclusive with respect to the weakened C=O double bond caused by the electron donation from the carbonyl unit to the scandium(III) cation, which is also evident in the UV–vis absorption of **MCM-ACR** (see below in Figure 9).

**Figure 3:**
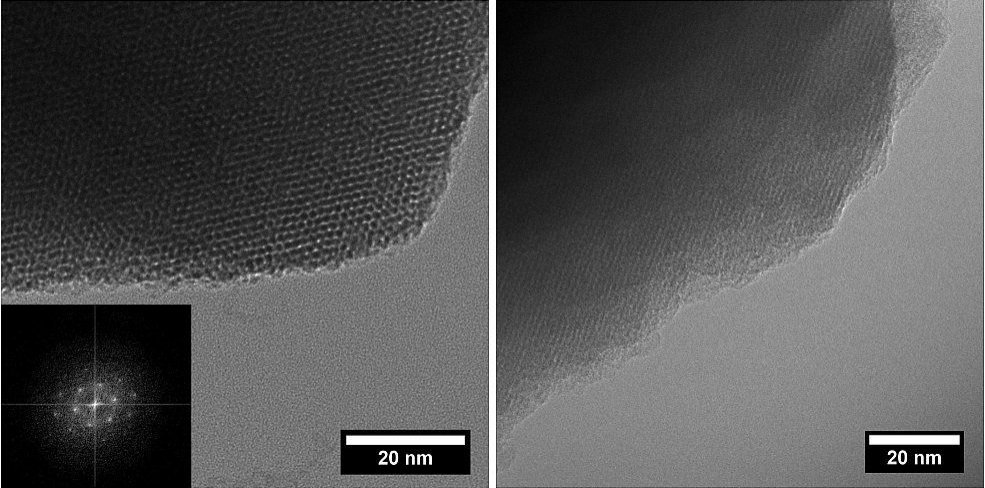
TEM images showing the mesoporous structure of **MCM-ACR** (left: frontal, right: lateral), inset in left image: Electron diffraction pattern.

**Figure 4:**
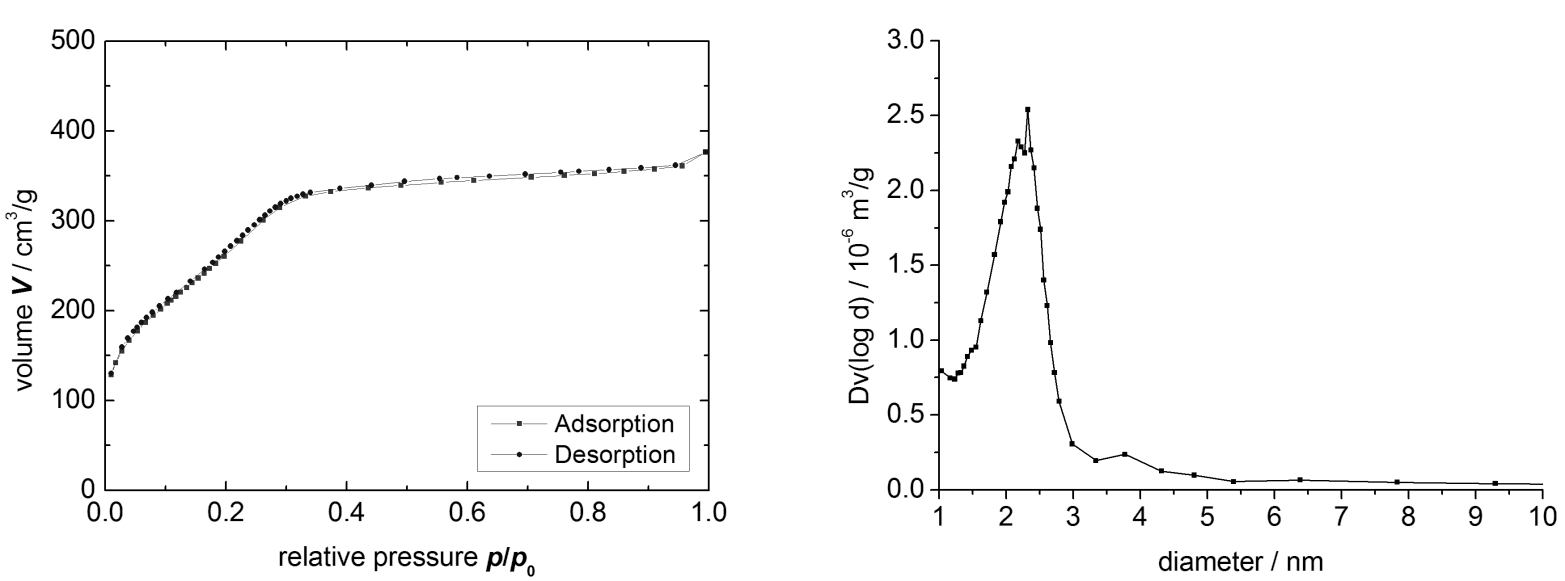
Sorption isotherm (left) and pore size distribution (BJH plot) (right) of **MCM-ACR**.

**Figure 5:**
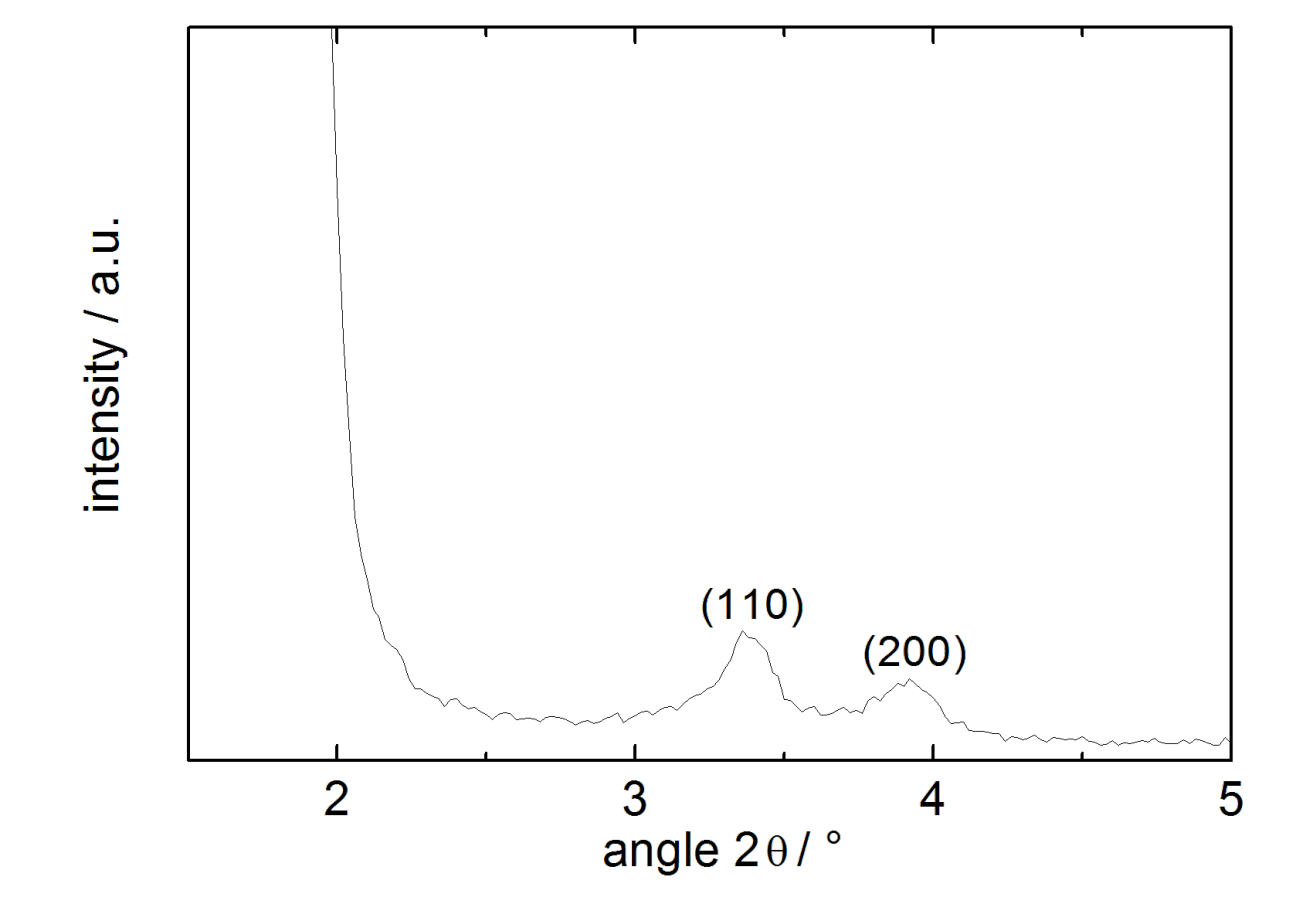
XRD pattern of **MCM-ACR**.

According to the TEM analysis (Figure 3) and BET measurements (Figure 4), the sol–gel process yielded a well ordered mesoporous material with a total surface area of up to 810 m^2^·g^−1^, exhibiting a characteristic pore size distribution with a sharp peak around 2.4 nm. The XRD spectrum reveals the expected peak patterns only for the (110) and (200) Miller indices as the (100) peak is not distinguishable from the primary beam (Figure 5). BET data of an authentic MCM-41 sample previously prepared in our group corresponded to a total surface area of 1122 m^2^·g^−1^, so the experimental value for **MCM-ACR** is in accordance with our expectations. Table 1 summarizes the values obtained for the described MCM-41-sample as well as for both Sc(III)-free and Sc(III)-containing **MCM-ACR**.

**Table 1:** BET and PSD date of different obtained mesoporous siliceous materials. In the case of **MCM-ACR**, two batches have been prepared with well reproducible outcomes (second batch in parentheses). The modified **MCM-ACR** sample was prepared from the first batch with a BET surface area of 775 m^2^·g^−1^.

type of material	BET surface [m^2^·g^−1^]	Langmuir surface [m^2^·g^−1^]	average pore diameter [nm]

MCM-41	1122	2346	2.4
**MCM-ACR**	775 (810)	1135 (1661)	2.4
**MCM-ACR** + Sc(OTf)_3_	734	962	2.3

The type IV BET isotherms of the material exhibit a large slope in the N_2_ uptake only at lower *p*/*p*_0_ ratios, and they show no significant sorption hysteresis. We may therefore conclude that the silica obtained has a very uniform structure and a homogeneous composition, thus proving that the co-condensation could be carried out in a controllable way, and that the formation of larger mesopores was prevented in the presence of the organic precursor in spite of its rather bulky nature.

**Figure 6:**
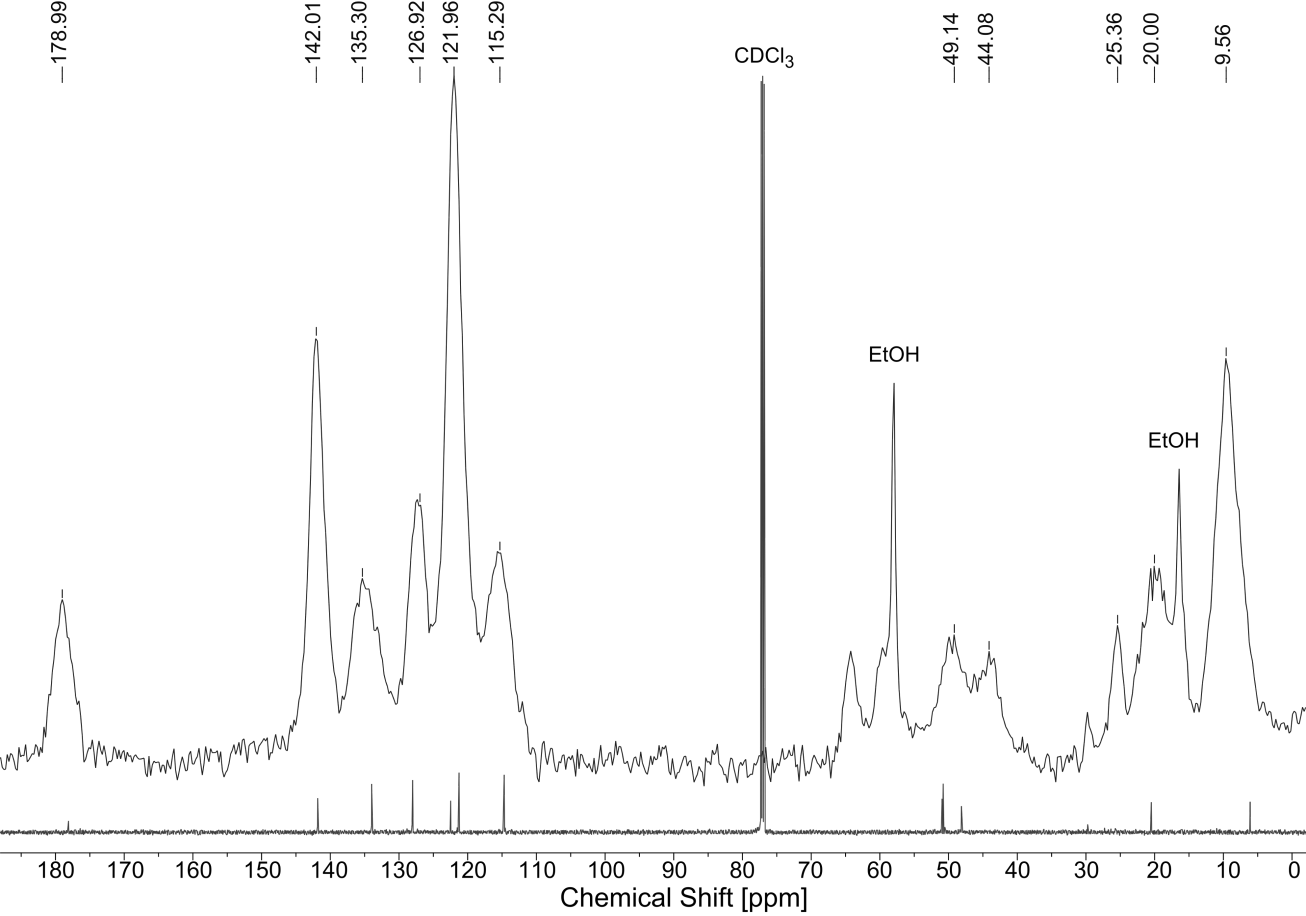
^13^C CP-MAS NMR spectrum of **MCM-ACR** overlaid with the high resolution ^13^C NMR spectrum of precursor **1** (recorded in CDCl_3_).

**Figure 7:**
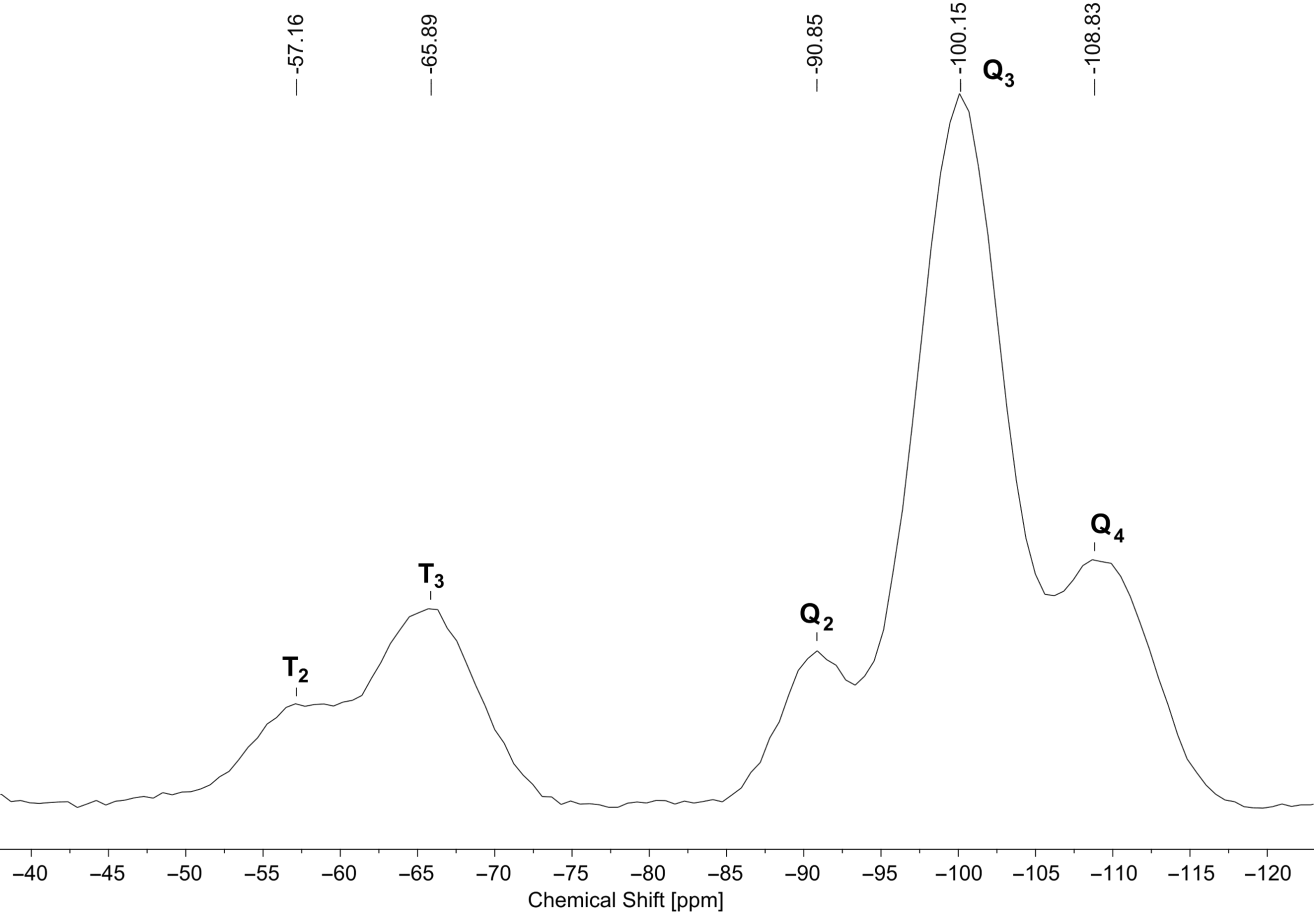
^29^Si CP-MAS NMR spectrum of **MCM-ACR**.

The ^13^C CP-MAS NMR spectrum of **MCM-ACR** (Figure 6) shows the expected signals for the three methylene groups of the propyl chain as well as for the acridone moieties. Minor impurities in the spectrum of the precursor are mostly due to hydrolysis occurring during the measurement. The two prominent sharp alkyl peaks in the solid state spectrum may be attributed to the remaining free EtOH within the silicate. The ^29^Si CP-MAS NMR data of **MCM-ACR** (Figure 7) prove that the material features the expected distribution of T- and Q-peaks. The minor T_2_-peak at −57 ppm can be ascribed to the R_alkyl_Si(OMe)(OSi)_2_ unit resulting from the partially incomplete incorporation of the precursor’s anchoring group into the framework [20].

**Figure 8:**
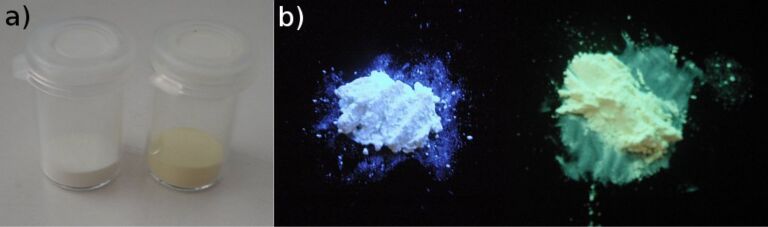
Visual appearance of **MCM-ACR** and **MCM-ACR** + Sc(OTf)_3_ under normal (a) and UV light (b).

**Figure 9:**
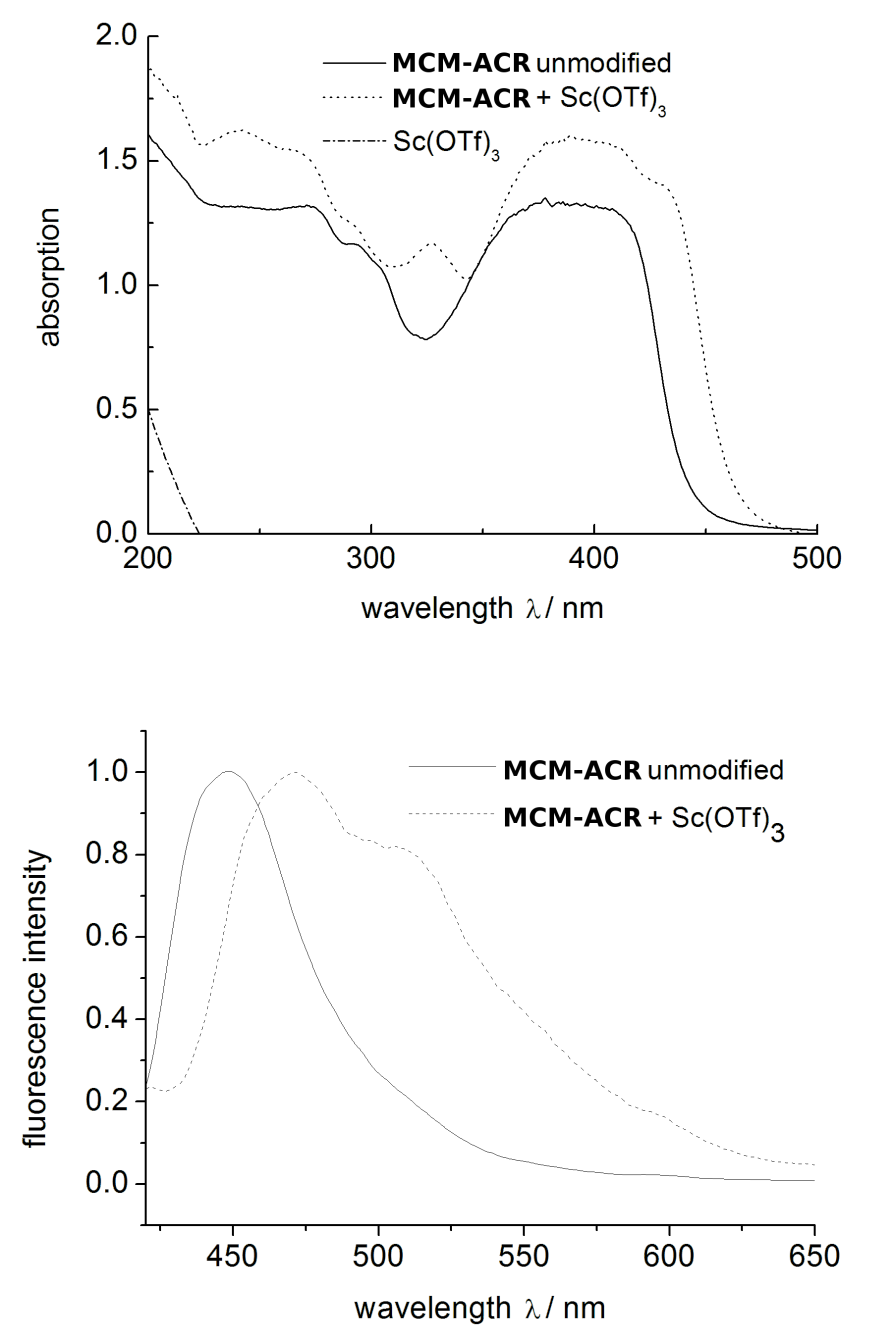
Overlay of the solid state UV-vis (top) and fluorescence (bottom) spectra of **MCM-ACR**, **MCM-ACR** + Sc(OTf)_3_ and pure Sc(OTf)_3_ (fluorescence excitation wavelength λ_ex._ = 400 nm). UV–vis-absorption intensities are not normalized to a given value; fluorescence has been normalized to an intensity of 1.0. Sc(OTf)_3_ shows no significant fluorescence and therefore was omitted in the second diagram.

When studying the fluorescence properties of the materials obtained before and after the post-sol–gel modification, an instantaneous shift in color and fluorescence was observed when **MCM-ACR** and the scandium(III) triflate solution were combined. Compound **1**, as well as unmodified **MCM-ACR**, feature a clear blue fluorescence when excited at a wavelength of λ_ex._ = 366 nm, whereas the complex formation with the scandium cation results in an intensely yellow colored product showing a greenish fluorescence (Figure 8). This effect is also clearly evident in the UV–vis and fluorescence spectra (Figure 9). Introduction of scandium(III) into the material yields a bathochromic effect in λ_abs._ and λ_em._ of about 20 nm and, more interestingly, a significantly increased absorption at around λ_abs._= 328 nm. The appearance of this distinct absorption may hint towards an electron transfer (LMCT) from the molecular orbital located at the carbonyl oxygen to the empty 3d orbital residing at the Sc(III) cation, a process which would be similar to those that have been described for carboxylate complexes of Eu(III) [21]. The fluorescence spectrum of the modified silica shows a distinct shoulder at around λ_em._≈ 515 nm. This indicates a complex radiative relaxation of the formed transition metal species, the fluorescence originating from at least two different transitions between the excited electronic states and the ground state. A reason for this phenomenon might be the energy splitting caused by the coupled CO stretching within the acridone–Sc^3+^ complex [7]. The pure triflate is almost completely transparent in the UV–vis region and shows no fluorescence when measured both as a solid film and in solution, hence it is obvious that the optical properties of the material depend on the electronic structure of the acridone chromophore, the latter being significantly changed via formation of the complex as the scandium(III) ion strongly interacts with the oxygen atom of the C=O functional group. The complex itself seems to be very stable and inert to ligand exchange: Excessive washing with polar organic solvents and storing of an authentic sample for weeks, and even months, neither altered the appearance of the material nor decreased its fluorescence intensity.

## Conclusion

We have presented a novel fluorescent organosilane bearing an acridone fluorophore and its successful transformation into a MCM-41 type material via co-condensation with TEOS. As predicted, the hybrid material shows a change in its fluorescence properties when non-covalently modified through scandium complex formation. The miscibility of the organic precursor or its concentrated solution with an excess of the major silicon source has been determined to be crucial for the synthesis of the hybrid material given herein. Possibilities to vary the dye content by using different molar ratios of the precursors in the sol–gel process are to be investigated in the near future. Furthermore, efforts will be made to elucidate the optical properties of the materials after doping with lanthanides or heavy metal cations, e.g. Eu(III), Er(III) or Bi(III).

## Experimental

**General:** All starting materials described herein were purchased from Sigma-Aldrich and used as received. Solvents for the organic syntheses were dried prior to use according to standard procedures [22]. Solid-state ^1^H CP-MAS, ^29^Si CP-MAS and ^13^C CP-MAS NMR spectra were recorded on a Bruker DSX Avance NMR spectrometer at resonance frequencies of 400 MHz, 101 MHz and 80 MHz for ^1^H, ^13^C or ^29^Si nuclei, respectively. Liquid phase ^1^H and ^13^C NMR spectra were recorded on Bruker Spectrospin DPX-400 and Avance 600 devices at resonance frequencies of 400 MHz or 151 MHz for ^1^H or ^13^C nuclei, respectively. These spectra are internally referenced to SiMe_4_. The infrared spectra with a resolution of ±2 cm^−1^ were recorded using a PerkinElmer FT-ATR-IR 1000 spectrometer containing a diamond coated ZnSe-window. MALDI-ToF measurements were conducted on a Bruker Daltonics Ultraflex spectrometer. Elemental analyses were determined on a CHNS vario Microcube elemental analyzer (Elementar). X-ray powder diffraction (PXRD) patterns of the silica samples were recorded on a Siemens D5005 instrument using Ni-filtered Cu Kα radiation (λ = 1.5404 Å), with a step size of 1 °/min. N_2_-Adsorption–desorption isotherms, pore size distributions as well as the textural properties of the hybrid materials were determined at 77 K by a Quantachrome Autosorb 1 sorption analyzer. Before analysis, the samples were activated at 120 °C overnight in the vacuum and then the adsorption–desorption procedure was conducted by passing nitrogen into the sample, which was kept under liquid nitrogen. The average pore size of the samples was estimated using the BJH approach based on the Kelvin equation while assuming a cylindrically shaped porous structure. The specific surface areas were calculated by means of the Brunauer–Emmett–Teller (BET) equation in the low relative pressure interval (<0.3) and the pore size distribution curves were analyzed with the adsorption branch by the BJH method. The morphology of the mesoporous particles was determined by a Philips CM 300 UT field emission transmission electron microscope (TEM) with 300 kV acceleration voltage and 0.17 nm point resolution. The UV–vis absorption and fluorescence of the precursor were measured using a Perkin–Elmer Lambda 900 and a Horiba Jobin–Yvon Fluorolog 3-22 τ in steps of 0.1 nm and 1.0 nm, respectively, the SiO_2_-cuvettes used had a width of 1.0 cm. Solid state UV–vis measurements were carried out on a Perkin–Elmer Lambda 18 double beam UV–vis spectrometer with double monochromator by setting the wavelength range from 200 nm to 900 nm in a 1 nm step width. The optical unit included a pre-aligned tungsten-halogen lamp and a deuterium lamp with automatic source exchange. All powder samples were calibrated with a diffuse BaSO_4_ referenced auto zero and were measured using a Biconical (Praying Mantis) Diffuse Reflectance Accessory in reflecting absorption mode. Solid state fluorescence data was recorded on a Perkin-Elmer LS55 with a step width of 0.5 nm. The thin film powder samples were prepared from a DCM suspension or solution by evaporation of the solvent on a glass substrate.

**10-(Trimethoxysilylpropyl)acridin-9(10H)-one** (**1**)**:** Finely powdered dry acridone [23] (5.85 g, 30.0 mmol) was added portionwise to a stirred suspension of 1.1 equiv of sodium hydride (800 mg, 33.3 mmol) in dry THF (100 mL). The resulting yellow-greenish suspension was stirred for 15 min at 25 °C until the hydrogen evolution subsided. Subsequently, 1.1 equiv of 3-iodopropyltrimethoxysilane (9.67 g, 6.53 mL, 33.3 mmol) were added dropwise via a syringe through a rubber septum. The golden colored suspension was heated to reflux for 24 h before evaporation of the solvent. The residue was washed with dry pentane (3 × 10 mL), then re-dissolved in several portions of dichloromethane (total of 150 mL) and the combined organic solutions were filtered. Evaporation of the solvent gave **1** as a highly viscous orange oil (16.9 mmol, 6.77 g, 56%) showing a strong blue–green fluorescence under UV light. ^1^H NMR (400 MHz, CDCl_3_) δ 8.61 (d, ^3^*J*_HH_ = 7.9 Hz, 2H), 7.76 (t, ^3^*J*_HH_ = 7.8 Hz, 2H), 7.59 (d, ^3^*J*_HH_ = 8.7 Hz, 2H), 7.32 (t, ^3^*J*_HH_ = 7.5 Hz, 2H), 4.51–4.34 (m, 2H), 3.67 (s, 9H), 2.22–2.01 (m, 2H), 0.89 (t, ^3^*J*_HH_ = 7.7 Hz, 2H); ^13^C NMR (151 MHz, CDCl_3_) δ 178.0, 141.8, 133.9, 127.9, 122.4, 122.2, 114.6, 50.8, 48.1, 20.4, 6.1; ATR-IR (ZnSe) 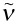 [cm^−1^]: 2940, 2840, 1635, 1599, 1491, 1462, 1378, 1342, 1291, 1264, 1169, 1045, 956, 937, 897, 810, 753, 673; MALDI-ToF for C_19_H_23_NO_4_Si (matrix CHCA, M^+^): 356.6; UV–vis (CH_2_Cl_2_, *c* ≈ 10^−6^ M) λ_abs._ = 254, 381, 400 nm; Fluorescence (CH_2_Cl_2_, *c* ≈ 10^−6^ M, λ_ex._ = 250 nm): λ_em._ = 415, 431 nm.

**Acridone functionalized hybrid silica material** (**MCM-ACR**)**:** The molar ratio of the different sol–gel components was determined in advance to be 9.0:1.0:24.0:1.4:1000 (TEOS:precursor **1**:base:C_18_TAB:H_2_O). In order to achieve a homogeneous co-condensation, the organic precursor ideally had to be dissolved prior to hydrolysis. An aqueous 70 wt % solution of H_2_NEt (12.2 g, 190 mmol) was introduced into a stirred solution of C_18_TAB (4.35 g, 11.1 mmol) in deionized water (142 mL). A two-phase mixture consisting of TEOS (14.8 g, 15.7 mL, 71.0 mmol) and **1** (3.15 g, 8.82 mmol) was rapidly added at 25 °C by syringe under vigorous stirring. The milky yellow solution, which soon contained a precipitate, was stirred at room temperature for 5 h before being heated to 100–110 °C (bath temperature) for a further 16 h. The resulting hot suspension was filtered, washed thoroughly with deionized water (a total amount of 1 L) and the solid residue was re-suspended in a 1:8 mixture of ethanol and concentrated HCl (200 mL). C_18_TAB was extracted by stirring for 16 h at 85 °C. The obtained solid was filtered from the hot solution, washed with EtOH (500 mL) and dried in the vacuum to give the product as a pale yellow, very fine powder (6.60 g, 7.76 mmol according to a calculated molecular weight of 835.7 g·mol^−1^). CHNS analysis found: C 21.25, H 3.56, N 1.66; calcd. for (C_16_H_14_NO_2.5_Si)·(H_2_O)_9_·(SiO_2_)_7.5_: C 21.33, H 3.48, N 1.55. This gives a CHN content of approx. 24 wt % (related to the *N*-propylacridone moiety).

**Post-sol–gel modification of MCM-ACR:** Introduction of the scandium salt into the material was realized by stirring **MCM-ACR** (500 mg) in a 0.01 M solution of scandium(III) triflate in ethanol or acetonitrile (25 mL) for 16 h. The modified materials were thoroughly washed with ethanol or acetonitrile (5 × 5 mL) and water (5 × 5 mL) prior to drying and characterization.
